# *Streptococcus pneumoniae* Cerebellar Abscess in a Child With Specific Polysaccharide Antibody Deficiency: A Case Report

**DOI:** 10.1155/crpe/4164740

**Published:** 2026-08-27

**Authors:** Barbara Anna Folga, Adam S. Bronson, Daniel Whorf, Joyce Rabbat, Lilly Immergluck

**Affiliations:** ^1^ Department of Pediatric Infectious Diseases, The University of Chicago Comer Children’s Hospital, Chicago Illinois, 60637, USA; ^2^ Loyola University Stritch School of Medicine, Maywood Illinois, 60153, USA, luc.edu; ^3^ Department of Pediatrics, Loyola University Medical Center, Maywood Illinois, 60153, USA, loyolamedicine.org; ^4^ Department of Pediatric Allergy and Immunology, Loyola University Medical Center, Maywood Illinois, 60153, USA, loyolamedicine.org

**Keywords:** brain abscess, case report, specific antibody deficiency, *Streptococcus pneumoniae*

## Abstract

Brain abscesses are local pockets of infection within the brain parenchyma, characterized by headaches, fevers, and focal neurological deficits. Here, we present a case of an eight‐year‐old fully vaccinated male patient who presented to the emergency department with a 1‐week history of intermittent generalized headache, photophobia, fatigue, and vomiting. The patient was admitted for further work‐up, with subsequent imaging revealing a cerebellar mass consistent with an abscess. Cultures from the abscess fluid grew pan‐sensitive *Streptococcus pneumoniae*Serotype 3, and the patient was started on a 6‐week course of antibiotic therapy, completed via an outpatient peripherally inserted central catheter (PICC) line. Considering that this serotype is one of the 13 serotypes included in the pneumococcal conjugate vaccine 13 (PCV13), potential etiologies for the patient’s presentation were proposed, including underlying immunodeficiency. An immunodeficiency work‐up diagnosed him with specific polysaccharide antibody deficiency (SPAD) with poor immunologic memory after vaccination against *Streptococcus pneumoniae,* and *Haemophilus influenzae* failed to induce robust and durable antibody levels. This case underscores the importance of screening children who were previously immunized with PCV and present with invasive pneumococcal disease (IPD) for underlying immunodeficiency.

## 1. Introduction

*Streptococcus pneumoniae* is a Gram‐positive, encapsulated diplococci belonging to the *Streptococcaceae* family [1]. Infections with this organism in the pediatric population range from mild, such as acute otitis media and sinusitis, to severe, with patients presenting with signs and symptoms of invasive pneumococcal disease (IPD) including meningitis, necrotizing pneumonia, osteomyelitis, and bacteremia. Brain abscesses represent a rare presentation of IPD [2]. IPD occurs most commonly in children under 5 years of age, especially in children under 2, due to an immature immune response in this age group, increased frequency of exposure, and propensity for bacterial colonization. Immunization against multiple serotypes using the conjugated pneumococcal vaccine has resulted in a significant decline in the number of IPD infections worldwide [3]; however, cases of IPD in fully vaccinated children and adults have been well documented in the literature [4–6].

## 2. Case Presentation

We present a case of an eight‐year‐old fully vaccinated male patient, who received 4 doses of pneumococcal conjugate vaccine 13 (PCV13) prior to 24 months of age, with a past medical history significant for omphalocele status post repair in infancy, as well as frequent upper respiratory infections every 3‐4 weeks, who presented to an outside emergency department (ED) with a 1‐week history of intermittent generalized headache, photophobia, fatigue, and vomiting. Approximately 1 week prior to the onset of these symptoms, the patient experienced a gastrointestinal infection, with emesis, fevers, and abdominal pain, which had since resolved. The patient and parents denied new‐onset fevers, recent travel history, and known immunodeficiencies.

**FIGURE 1:**
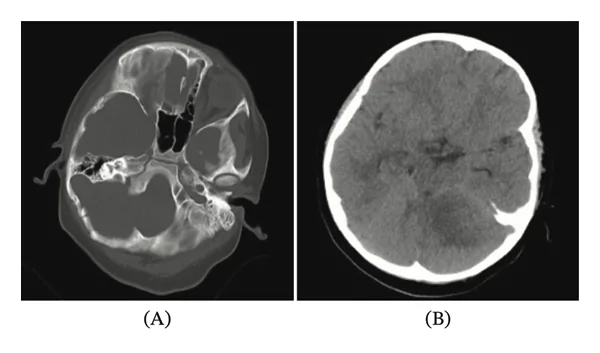
CT head without IV contrast. (A, B) Mass within the left cerebellar hemisphere with associated vasogenic edema. There is no associated herniation or hydrocephalus.

Upon arrival at the outside hospital ED, the patient’s vital signs were stable, and he exhibited a reassuring neurological examination. A complete blood count revealed a mild leukocytosis of 13 K/μL (86% segmented neutrophils, 10% lymphocytes, and 4% monocytes). Due to the nature of the patient’s clinical condition, a computerized tomography (CT) scan of the head was obtained, demonstrating a mass within the left cerebellar hemisphere, with associated vasogenic edema and without associated herniation or hydrocephalus (Figures 1A and B). Despite this mass and risk of cerebellar/tonsillar herniation, the patient remained neurologically stable. Based on these imaging findings, the patient was transferred to our healthcare facility, a quaternary care center with a pediatric intensive care unit (PICU), for a higher level of care.

**FIGURE 2:**
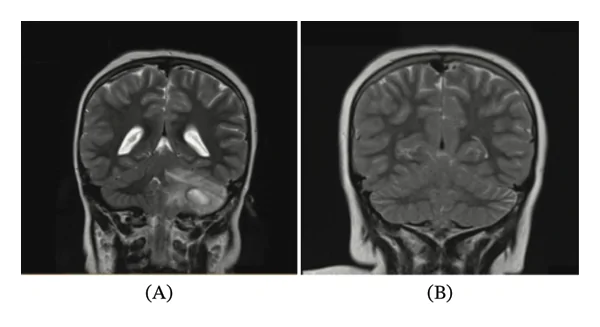
MRI head with (left) and without (right) IV contrast. (A) Approximately 2 cm, irregularly enhancing, cystic mass within the left cerebellar hemisphere with associated surrounding edema, regional mass effect, and approximately 6 mm midline shift. The findings are nonspecific, especially given the lack of clinical and serological markers for infection, but include infection, an inflammatory process, or neoplasm, including a variant appearance for pilocytic astrocytoma. (B) Resolution of the left cerebellar abscess 2.5 months after completion of the antibiotic course.

Upon arrival to the PICU, he was observed to be in distress, wincing and tightly shutting his eyes, complaining of a headache and photophobia. Pediatric neurosurgery recommended obtaining magnetic resonance imaging (MRI) of the head with intravenous contrast (Figure 2A). Due to concerns for a possible infectious process, the pediatric infectious disease (ID) team was consulted for further management. On hospital day 2, the patient went to the operating room (OR) for a left‐sided burr hole placement for stereotactic drainage of a suspected abscess. While in the OR, fluid from the lesion was sent for routine studies including a Gram stain, aerobic and anaerobic bacterial cultures, and fungal culture. Shortly after the procedure, the patient was started on empiric ceftriaxone, vancomycin, and metronidazole. Culture results returned positive for *Streptococcus pneumoniae*; antibiotic susceptibility testing indicated the organism was susceptible to penicillin and pan‐sensitive to all antibiotic classes tested. Based on these results, vancomycin and metronidazole therapies were discontinued. The patient’s bacterial isolate was submitted to the Centers for Disease Control and Prevention (CDC) for serotyping, which identified the organism as *Streptococcus pneumoniae* Serotype 3. Serotyping was undertaken because the patient had verified documentation of having received all four recommended doses of PCV13, with the final dose administered in 2017. Since Serotype 3 is included within PCV13, the patient’s serum was collected to quantify *Streptococcus pneumoniae* serotype–specific immunoglobulin G (IgG) concentrations. Interestingly, initial levels demonstrated a high Serotype 3‐specific antibody concentration (Table 1). Considering that this serotype is covered by immunization, potential etiologies for the patient’s presentation were proposed, including an underlying humoral immunodeficiency causing an inadequate response to vaccination, with a further work‐up dictated by pediatric allergy and immunology as an outpatient.

**TABLE 1:** *Streptococcus pneumoniae* serotype–specific IgG, *Haemophilus influenzae* Type b IgG, and total quantitative immunoglobulin concentrations.

**Immunoglobulin G (IgG) concentration (mcg/mL)**					

**Serotype**	**Reference range (mcg/mL)**	**01/17/2024 (hospitalization)**	**05/22/2024 (1 month post-pneumovax)**	**07/22/2024 (1 month post-pedvaxHib)**	**02/03/2025**

1	> 1.3	< 0.3	0.8	—	0.2
3	> 1.3	**> 44.0**	0.6	—	0.3
4	> 1.3	< 0.3	**2.3**	—	0.4
5	> 1.3	**1.2**	0.6	—	0.1
8	> 1.3	0.4	**17.2**	—	**4.2**
9 (9N)	> 1.3	< 0.3	**16.1**	—	**3.8**
12 (12F)	> 1.3	< 0.3	0.2	—	0.2
14	> 1.3	0.4	**4.7**	—	**2.5**
19 (19F)	> 1.3	**1.3**	**2.3**	—	0.7
23 (23F)	> 1.3	1.1	**> 31.2**	—	**8.4**
26 (6B)	> 1.3	< 0.3	**4.0**	—	1.1
51 (7F)	> 1.3	< 0.3	0.7	—	< 0.1
56 (18C)	> 1.3	< 0.3	0.5	—	< 0.1
68 (9V)	> 1.3	< 0.3	**2.0**	—	0.4
*H. influenzae* Type b	> 1.0	—	< 0.11	**> 9.0**	0.36

**Serum immunoglobulin concentration by isotype (mg/dL)**					

**Isotype**	**Reference range (mg/dL)**	**Hospitalization**	**1 month postpneumovax**	**6 months posthospitalization**	**11 months postpneumovax**

Immunoglobulin G	510–1260	1318 (H)	—	—	—
Immunoglobulin A	50–230	207	—	—	—
Immunoglobulin M	40–130	128	—	—	—

*Note:* Bold denotes adequate antibody levels. Pneumovax was given on 04/18/2024, and PedvaxHib was given on 06/18/2024.

Throughout the remainder of his hospital course, the patient’s pain was well‐controlled, and he maintained stable vitals without fevers. A peripherally inserted central catheter (PICC) line was placed on day 4 of his hospitalization, and he was discharged on hospital day 8 on meningitic dosing of ceftriaxone as an outpatient for a total of 6 weeks of therapy, with close follow‐up with both the pediatric ID and immunology teams.

Figure 2B illustrates imaging findings approximately 2.5 months after completion of a 6‐week course of ceftriaxone, showing complete resolution of the abscess. Given the patient’s diagnosis of IPD and the low antibody levels against *Streptococcus pneumoniae* and *H. influenzae* Type b (Hib) despite completing the 4‐dose vaccination series in 2017 (Table 1), the pediatric immunology team performed diagnostic immunizations with Pneumovax 23 (unconjugated form of vaccine targeting 23 serotypes of *S. pneumoniae*) and Hib, followed by repeat antibody levels 4–6 weeks postvaccination to assess the patient’s antibody response to vaccinations.

Though the definition of adequate antibody response is controversial, since the patient was vaccinated with Pneumovax 23 (rather than Prevnar), an IgG threshold of > 1.3 mcg/mL was considered an adequate response following vaccination [7]. One month following vaccination with Pneumovax 23, antibody levels demonstrated adequate coverage for 8 out of the 14 serotypes tested (Table 1). Interestingly, however, the IgG titer to Serotype 3 had dropped from > 44 mcg/mL to 0.6 mcg/mL in just 4 months following IPD by *S. pneumoniae* Serotype 3. This finding indicated poor memory. Approximately 11 months after vaccination, antibody levels to all *S. pneumoniae* serotypes and Hib dropped (Table 1), prompting a diagnosis of specific polysaccharide antibody deficiency (SPAD) with a poor immunologic memory phenotype. Complement studies were normal. A primary immunodeficiency gene panel using next‐generation sequencing was completed, which did not detect any pathogenic variants. It should be noted that there was no family history of inborn errors of immunity. Prior to presentation, the patient suffered from recurrent nonfebrile upper respiratory infections every 3‐4 weeks that were not treated with antibiotics, along with numerous episodes of acute otitis media in his lifetime. Following the diagnosis of SPAD (memory phenotype), the patient was started on subcutaneous IgG replacement therapy every 2 weeks, which he has tolerated well. Since starting IgG replacement therapy, his recurrent upper respiratory infections have resolved, and he has had no further invasive infections. Tentative plans include continuing the infusions for at least the next 1–2 years, with re‐evaluation of his immune profile at that time.

## 3. Discussion

Brain abscesses in children characteristically present with headaches, fevers, and focal neurologic deficits. In a retrospective review conducted by Gilard and colleagues, Gram‐positive cocci were found to be the leading cause of brain abscesses, with streptococci being the most common [8]. Other frequently isolated microorganisms included Gram‐negative bacilli, anaerobic bacteria, *Mycobacterium* species, and *Aspergillus* species [9]. While rare in children, brain abscesses are more likely to occur and represent a significant risk for immunocompromised individuals [10]. Our case of an eight‐year‐old previously healthy patient is intriguing in light of the fact that he is older than 5 years of age and showed evidence of complete and timely immunization against *S. pneumoniae* before the age of 2 years. Most documented cases of IPD occur in those less than 5 years of age and among those partially immunized.

The patient in our case presented with several features characteristic of an underlying brain abscess, including headache, photophobia, and fatigue, yet did not have an obvious predisposing risk factor. He was diagnosed with SPAD, a primary immunodeficiency characterized by normal concentrations of total serum immunoglobulins and normal responses to protein antigens, but inadequate responses to polysaccharide antigens (Table 1), often predisposing patients to severe and/or recurrent infections by encapsulated bacteria [11, 12]. Curiously, Serotype 3‐specific IgG antibodies were elevated in our patient during his hospitalization despite the definitional inability to mount an antibody response to polysaccharide antigens. This is not completely unexpected as pneumococcal polysaccharide capsule antigens are associated with immunogenic protein antigens, enabling a B/T‐cell cooperative response during infection. In brief, pneumococcal polysaccharide antigens associated with protein antigens bind the B‐cell receptor, are internalized and processed, and protein‐derived peptides are presented to T‐cells via MHCII, a process that is largely preserved in SPAD [13]. The inability to sustain a memory response to Pneumovax 23, an unconjugated (contains no protein) polysaccharide vaccine, is consistent with the memory phenotype of SPAD.

In a prospective study by Stabler and colleagues, adults with recurrent and severe bacterial infections were screened for primary immunodeficiencies, including SPAD. Of 118 screened, 47 (39.8%) were diagnosed with a primary immunodeficiency. Of these 47 patients, 37 (78.7%) were diagnosed with SPAD after failing to mount an appropriate immune response to pneumococcal polysaccharide vaccines [6]. A systematic review of > 6000 children over the age of two years, diagnosed with recurrent infection and/or IPD, demonstrated a frequency of primary immunodeficiency between 1.3% and 10.5%. Of these primary immunodeficiencies, antibody deficiency was most common, suggesting that SPAD is a relatively common comorbidity in patients with recurrent infections and/or IPD [14]. Management for SPAD includes a combination of prophylactic antibiotic therapy, repeated vaccination, and IgG replacement, with antibiotic therapy and IgG replacement therapy being demonstrated to be equally efficacious [12, 15, 16].

*Streptococcus pneumoniae* Serotype 3, the serotype our patient was infected with, behaves differently than other serotypes, owing largely to its thick, mucoid polysaccharide capsule that has been demonstrated to impair opsonization and phagocytosis in the host, contributing to this pathogen’s high virulence [17]. Notably, Serotype 3 has remained a frequent cause of breakthrough pneumococcal disease even in vaccinated individuals, suggesting that current pneumococcal conjugate vaccines stimulate poor functional antibody responses toward Serotype 3 [18, 19]. Because these vaccine failures contribute to substantial morbidity and mortality—and because the immunologic and epidemiologic factors underlying them remain incompletely characterized in preadolescents and adolescents—further investigation is warranted.

This case emphasizes the importance of evaluating fully immunized children presenting with invasive bacterial disease, particularly by encapsulated organisms (pneumococci, Hib, meningococci, etc.), for immunodeficiency, including SPAD, so that appropriate counseling can be offered to patients and their families to minimize risk of further invasive bacterial infections [20]. This case also stresses that continued efforts be made toward appropriate preventative vaccination in all eligible children.

## Funding

No funding was received for this study.

## Conflicts of Interest

The authors declare no conflicts of interest.

## Supporting Information

Additional supporting information can be found online in the Supporting Information section.

## Supporting information


**Supporting Information** CARE checklist SPAD.

## Data Availability

Data sharing is not applicable to this article as no datasets were generated or analyzed during the current study.
