# Supplementary material for: Streptococcus pneumoniae Cerebellar Abscess in a Child With Specific Polysaccharide Antibody Deficiency: A Case Report
Source: Case Rep Pediatr. 2026 Aug 27;2026:4164740. doi: 10.1155/crpe/4164740 (PMC13522628; doi:10.1155/crpe/4164740)
Supplement: Supplementary file 1 [file CRPE-2026-4164740-s001.pdf]

CARE
case report guidelines

CARE Checklist of information to include when writing a case report

(cc) BY-NC-ND

Topic	Item	Checklist item description	Reported on Line
Title	1	The diagnosis or intervention of primary focus followed by the words "case report"	1-2
Key Words	2	2 to 5 key words that identify diagnoses or interventions in this case report, including "case report"	54
Abstract (no references)	3a	Introduction: What is unique about this case and what does it add to the scientific literature?	51-53
	3b	Main symptoms and/or important clinical findings	39-43
	3c	The main diagnoses, therapeutic interventions, and outcomes	44-45
	3d	Conclusion—What is the main "take-away" lesson(s) from this case?	51-53
Introduction	4	One or two paragraphs summarizing why this case is unique (**may include references**)	192-197
Patient Information	5a	De-identified patient specific information.	69-72
	5b	Primary concerns and symptoms of the patient.	73-74
	5c	Medical, family, and psycho-social history including relevant genetic information	76-77
	5d	Relevant past interventions with outcomes	—
Clinical Findings	6	Describe significant physical examination (PE) and important clinical findings.	87-88
Timeline	7	Historical and current information from this episode of care organized as a timeline	69-113
Diagnostic Assessment	8a	Diagnostic testing (such as PE, laboratory testing, imaging, surveys).	89, 92, 100
	8b	Diagnostic challenges (such as access to testing, financial, or cultural)	—
	8c	Diagnosis (including other diagnoses considered)	105-107
	8d	Prognosis (such as staging in oncology) where applicable	114-115
Therapeutic Intervention	9a	Types of therapeutic intervention (such as pharmacologic, surgical, preventive, self-care)	92, 112
	9b	Administration of therapeutic intervention (such as dosage, strength, duration)	136-137
	9c	Changes in therapeutic intervention (with rationale)	—
Follow-up and Outcomes	10a	Clinician and patient-assessed outcomes (if available)	138-139
	10b	Important follow-up diagnostic and other test results	125-129
	10c	Intervention adherence and tolerability (How was this assessed?)	138-141
	10d	Adverse and unanticipated events	—
Discussion	11a	A scientific discussion of the strengths AND limitations associated with this case report	—
	11b	Discussion of the relevant medical literature **with references**.	154-191
	11c	The scientific rationale for any conclusions (including assessment of possible causes)	156-158
	11d	The primary "take-away" lessons of this case report (without references) in a one paragraph conclusion	196-197
Patient Perspective	12	The patient should share their perspective in one to two paragraphs on the treatment(s) they received	N/A
Informed Consent	13	Did the patient give informed consent? Please provide if requested	Yes ☐ No ☒
